# High-Throughput Screening of the Repurposing Hub Library to Identify Drugs with Novel Inhibitory Activity against *Candida albicans* and *Candida auris* Biofilms

**DOI:** 10.3390/jof9090879

**Published:** 2023-08-27

**Authors:** Olabayo H. Ajetunmobi, Gina Wall, Bruna Vidal Bonifacio, Lucero A. Martinez Delgado, Ashok K. Chaturvedi, Laura K. Najvar, Floyd L. Wormley, Hoja P. Patterson, Nathan P. Wiederhold, Thomas F. Patterson, Jose L. Lopez-Ribot

**Affiliations:** 1 Department of Molecular Microbiology & Immunology, South Texas Center for Emerging Infectious Diseases, The University of Texas at San Antonio, San Antonio, TX 78249, USA; olabayobebeyo@gmail.com (O.H.A.); ashok.chaturvedi@utsa.edu (A.K.C.); 2 Department of Healthcare Sciences, University of The Incarnate Word, San Antonio, TX 78209, USA; lamart13@uiwtx.edu; 3 Department of Medicine, Division of Infectious Diseases, The University of Texas Health Science Center at San Antonio, San Antonio, TX 78229, USA; najvar@uthscsa.edu (L.K.N.); patterson@uthscsa.edu (T.F.P.); 4 Department of Biology, Texas Christian University, Fort Worth, TX 76129, USA; floyd.wormley@tcu.edu; 5 Department of Pathology and Laboratory Medicine, The University of Texas Health Science Center at San Antonio, San Antonio, TX 78229, USA; pattersonhp@uthscsa.edu (H.P.P.); wiederholdn@uthscsa.edu (N.P.W.)

**Keywords:** *Candida* spp., biofilm, repurposing, screening, antifungal

## Abstract

Candidiasis is one of the most frequent nosocomial infections affecting an increasing number of at-risk patients. *Candida albicans* remains the most frequent causative agent of candidiasis, but, in the last decade, *C. auris* has emerged as a formidable multi-drug-resistant pathogen. Both species are fully capable of forming biofilms, which contribute to resistance, increasing the urgency for new effective antifungal therapies. Repurposing existing drugs could significantly accelerate the development of novel therapies against candidiasis. Here, we have screened the Repurposing Hub library from the Broad Institute, containing over 6000 compounds, in search for inhibitors of *C. albicans* and *C. auris* biofilm formation. The primary screen identified 57 initial hits against *C. albicans* and 33 against *C. auris*. Confirmatory concentration-dependent assays were used to validate the activity of the initial hits and, at the same time, establish their anti-biofilm potency. Based on these results, ebselen, temsirolimus, and compound BAY 11-7082 emerged as the leading repositionable compounds. Subsequent experiments established their spectrum of antifungal activity against yeasts and filamentous fungi. In addition, their in vivo activity was examined in the murine models of hematogenously disseminated *C. albicans* and *C. auris* infections. Although promising, further in vitro and in vivo studies are needed to confirm their potential use for the therapy of candidiasis and possibly other fungal infections.

## 1. Introduction

Infections caused by opportunistic pathogenic fungi within the genus *Candida* represent an increasing threat to an expanding population of immune- and medically compromised patients [1,2]. The limited number of antifungal drugs currently available for the treatment of candidiasis, their limited efficacy, and the emergence of resistance contribute to the high morbidity and mortality rates associated with candidiasis [3]. Approximately 50% of these infections are caused by *Candida albicans*, but, in recent years, the epidemiology of candidiasis is changing, as infections caused by non-*albicans Candida* species (NACS) are becoming increasingly common [4]. One NACS, in particular, has recently made headlines for its emergence as a formidable nosocomial pathogen. In 2009, *Candida auris* was first discovered in an ear infection in Japan [5]. Since then, this opportunistic fungal species has spread simultaneously across multiple continents and has caused outbreaks in several hospitals and healthcare facilities [6,7]. Notably, *C. albicans* and *C. auris* are among the fungal priority pathogens identified by the World Health Organization (WHO) as having the greatest threat to public health [8,9].

Both *C. albicans* and *C. auris* are fully capable of forming biofilms, which contribute to augmented antifungal resistance, as well as resistance to immunological assaults within the human body [10,11,12]. Given the levels of resistance and high levels of mortality detected with *Candida* infections associated with a biofilm etiology, it is clear that new antifungal options are desperately needed [3,13].

Repurposing (or repositioning) is the process of finding new therapeutic indications for current existing drugs, which can significantly decrease the time and effort in bringing drugs with novel antifungal activity from the bench to the bedside [14]. In the last decade, this approach has been fueled by the availability of repurposing libraries from different sources which can be used in high-throughput screenings, thereby facilitating the rapid identification of bioactive drugs in a variety of screens for different disease models, including those with antifungal activity [15]. The Broad Institute has recently created the Repurposing Hub, a comprehensive repurposing library and accompanying interactive and curated database [16], consisting of over 6000 compounds, many of which have been approved by the United States Food and Drug Administration (US FDA), and others which are at different stages of clinical development. Here, we report on the high-throughput screening of this chemical library in search for inhibitors of both *C. albicans* and *C. auris* biofilm formation.

## 2. Materials and Methods

### 2.1. Strains, Cultivation Conditions, and Media

*C. albicans* SC5314 and *C. auris* strain 0390, obtained from the U.S. Centers for Disease Control and Prevention (CDC), were used in this study, including primary screens and follow-up experiments. Working cultures were prepared on yeast extract–peptone–dextrose (YPD) (1% (*w*/*v*) yeast extract, 2% (*w*/*v*) peptone, and 2% (*w*/*v*) dextrose) agar plates. From these, the strains were grown by inoculating a loopful of cells in 20 mL of YPD liquid medium in 150 mL flasks and incubating in an orbital shaker at 30 °C overnight. The cells were washed with phosphate-buffered saline (PBS), counted, and adjusted to a final desired final cell density of 2 × 10^6^ cells/mL by diluting in RPMI-140 medium (without sodium bicarbonate, supplemented with L-glutamine, buffered with 165 mM morpholine propane sulfonic acid, and adjusted to pH 6.9). From now on, this medium will be referred to simply as “RPMI medium”.

### 2.2. Chemical Library

The Repurposing Hub library (Broad Institute, Cambridge, MA, USA) consists of about 6000 different compounds with 663 different therapeutic indications and over 2000 different targets [16]. Drugs in this collection are approved by the US FDA or have undergone testing in at least one phase of clinical trials [16]. Because of this, pharmacodynamics, pharmacokinetics, safety, and toxicity for humans have been characterized for most of these compounds.

### 2.3. High-Throughput Screen for Inhibitors of C. albicans and C. auris Biofilm Formation

The screening process was based on our previously described 96-well micotiter plate model of *Candida* biofilm formation [17,18], adapted to the use of 384-well microtiter plates to allow for true high-throughput screening [19]. Briefly, compounds in the Repurposing Hub library were pre-spotted by the Broad Institute in individual wells of 384-well flat-bottom microtiter plates (Corning Incorporated, Corning, NY, USA). Each well contained nanoliter volumes of one individual compound in the library, calculated in a manner that the addition of the cell suspension would result in a final screening concentration of 20 μM. Plates were bar-coded for identification purposes and were shipped to our laboratory as “assay-ready” plates to speed up the screening process. Upon receipt, plates with pre-spotted compounds were stored in our laboratory at −20 °C. In its final format, wells in columns 2 through 22 contained individual pre-spotted compounds, while columns 1 and 24 contained an equivalent volume of DMSO, and selected wells in column 23 contained Amphotericin B (at a final concentration of 4 μg/mL) as a positive control for inhibition. On the day of screening, plates were allowed to thaw and 30 μL of the cell suspension at a concentration of 2 × 10^6^ cells/mL in RPMI medium were added to individual wells in columns 2 through 24, while column 1 served as the sterility control and had only RPMI added. The plates were then incubated at 37 °C for 24 h to allow for biofilm formation. Then, the plates were washed once with 40 µL of PBS per well, and, after washing, 30 µL per well of XTT (2,3-bis(2-methoxy-4-nitro-5-sulfo-phenyl)-2H-tetrazolium-5-carboxanilide)/menadione solution were added, after which the plates were incubated in the dark for 1 h. The plates were then read for absorbance in a microtiter plate reader at 490 nm (from the top) to provide a quantitative measure of inhibition. The data were then normalized and analyzed in comparison with the growth control (in the absence of drug) to determine the percentage of inhibition. An arbitrary threshold of >70% biofilm inhibition was selected in order to identify initial “hits”.

### 2.4. Concentration-Dependent Assays for Confirmation of Initial Hits and Determination of Their Inhibitory Potency

Concentration-dependent measurements were carried out to reconfirm the activity of initial hits and to assess their potency. We used the same 384-well microtiter plate model for inhibition of *Candida* biofilm formation, but using a series of concentrations for each individual hit, which were pre-spotted by the Broad Institute prior to shipment to our laboratory as assay-ready plates. Briefly, wells in columns 3 to 22 contained 10-point concentration series of the different hits, in two-fold serial dilutions ranging from 40 µM to 0.078 µM final concentrations, allowing for up to 28 compounds to be tested in a single 384-well microtiter plate. The first column remained empty (to serve as media-only control), while columns 2 and 24 served as growth controls (no inhibition), and selected wells in column 23 contained Amphotericin B (as positive control for inhibition). Then, 30 µL of the standardized yeast cell inoculum were added to columns 2 through 24. After incubation at 37 °C for 24 h to allow for biofilm formation, the plates were washed once with PBS incubated with XTT and read at 490 nm in a microplate reader. Similar to the initial screen, the colorimetric readings were analyzed to determine the percentage of inhibition for each drug at the 10 different concentrations. From these results, the inhibitory concentration required to inhibit 50% of growth (IC_50_) was determined by fitting normalized results (positive (untreated) and negative (uninoculated) controls arbitrarily set as 100% and 0% growth) to the variable slope Hill equation (an equation that determines the nonlinear drug dose–response relationship) using Prism (version 10.0.2, GraphPad Software Inc., San Diego, CA, USA). Compounds found to inhibit greater than 70% of biofilm growth in the dilution series were considered to be confirmed “hits”.

### 2.5. Antifungal Susceptibility Testing to Determine the Spectrum of Activity of the Leading Repositionable Drugs Ebselen, Temsirolimus, and BAY 11-7082 against a Panel of Medically Important Fungi

Antifungal susceptibility testing was performed using standard CLSI methods, to examine the activity of the selected leading repositionable compounds ebselen, temsirolimus, and BAY 11-7082 against a panel of medically important fungi, including yeasts and molds. All clinical fungal isolates tested form part of the collection available in the Fungus Testing Laboratory at the University of Texas Health Science Center at San Antonio. MICs were determined in accordance with the CLSI M27 (for yeast) and M38 (for filamentous fungi) reference standards for antifungal susceptibility testing [20,21]. Stock solutions of resupplied ebselen, temsirolimus, and BAY 11-7082 were prepared by dissolving the powders in DMSO. Further dilutions were prepared in RPMI medium. Fluconazole (for yeasts), and posaconazole or voriconazole (for molds) were used for comparison purposes. The minimum inhibitory concentrations (MICs) were read visually at 50% and/or 100% of growth after 24 to 72 h of incubation for yeasts and filamentous fungi depending upon the species tested against.

### 2.6. Preliminary Examination of the In Vivo Antifungal Activity of the Leading Repositionable Compounds Ebselen, Temsirolimus, and BAY 11-7082 in Murine Models of Hematogenously Disseminated Candidiasis

All animal experiments were performed following NIH guidelines and in accordance with institutional regulations (IACUC) in AAALAC-certified facilities. Animals were randomly distributed in different cages and allowed a one-week acclimatization period before experiments were started. Throughout the studies, mice were observed multiple times per day to prevent and minimize unnecessary pain and distress that may have occurred with infection. Any animal that appeared moribund was humanely euthanized. Persons monitoring the animals were not blinded as to the identity of the different groups.

The initial assessment of the antifungal activity in vivo of ebselen, temsirolimus, and compound BAY 11-7082 used the well-established model of hematogenously disseminated *C. albicans* infections, and was performed following methodologies previously described by our group. Briefly, cultures of *C. albicans* SC5314 strain were grown overnight in YPD broth at 25 °C. Cells were harvested by centrifugation, washed, counted, and diluted appropriately in sterile saline for injection to prepare the infecting inoculum. Then, a final volume of 200 µL containing 3.5 × 10^5^ yeast cells was injected via the lateral tail vein into 6- to 8-week-old female BALB/c mice. The drugs were diluted in 2% DMSO and prepared in saline for injection, with doses of 2.5 mg/kg for temsirolimus and 5 mg/kg for both ebselen and BAY 11-7082 administered intraperitoneally to groups of mice (n = 8). In order to maximize the detection of protective effects, we used a prophylactic regimen with treatment starting 2 days prior to infection, and then continuing once daily until the end of the observational period (typically 14 days). A control group was on the same schedule but received vehicle-only injections.

The *C. auris* infection model has been previously described [22,23]. Briefly, male ICR mice (10 per group) were rendered neutropenic with a single dose of pharmaceutical-grade 5-fluorouracil (5 mg/mouse) administered 24 h prior to inoculation. To prevent bacterial superinfection and deaths in the immunosuppressed mice, mice received antibacterial prophylaxis consisting of enrofloxacin at 50 ppm in their drinking water beginning 1 day prior to infection. On the day of inoculation (day 0), a clinical isolate of *C. auris* (strain DI 17-46) was used to infect mice via the lateral tail vein (0.2 mL of a yeast cell inoculum of 1 × 10^7^ cells/mouse). Treatment groups consisted of vehicle control (2% DMSO), ebselen at 5 mg/kg, and temsirolimus at 2.5 mg/kg. Drugs were administered once daily by intraperitoneal injection, starting 2 days prior to infection until day 7 post-infections. Animals were monitored for a total of 21 days post-infection.

To determine the survival curves, days on which the mice died were recorded; for euthanized mice, death was recorded as occurring the next day. Survival was plotted by Kaplan–Meier analysis and differences between groups (treated versus untreated) were analyzed using the log rank test. Analyses were performed using Prism (GraphPad Software, Inc.).

## 3. Results and Discussion

### 3.1. High-Throughput Screening of the Drug Repurposing Hub for Inhibitors of C. albicans and C. auris Biofilm Formation

**Figure 1:**
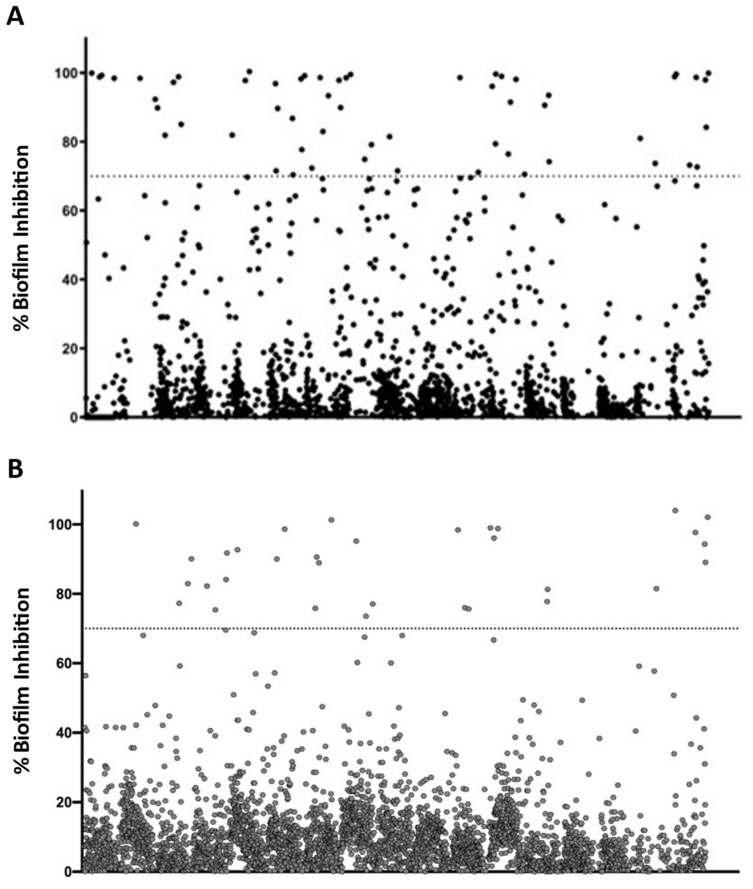
Graphical representation of results from the initial screens for inhibitors of biofilm formation against *C. albicans* (**A**) and *C. auris* (**B**). The dotted lines indicate the 70% arbitrary threshold for initial hit identification.

Repurposing represents an auspicious alternative for accelerated drug development, as exploring potential therapeutic utility in indications outside those originally targeted may drastically reduce the effort, time, and money required [14]. This approach is particularly attractive in disease areas with high unmet needs and a paucity of new leads, including antifungal drug development [13]. Most recently, repurposing efforts have been facilitated by the availability of repurposing screening libraries assembled by commercial entities and different organizations [15]. The chemical diversity and known safety profiles of drugs in these libraries (with most having been previously tested in humans) make this a particularly appealing approach with the potential to rapidly advance a candidate into the clinic. To address the shortage of antifungal drugs, particularly those with anti-biofilm activity [3,24], we screened the Repurposing Hub library provided by the Broad Institute [16] in search for inhibitors of biofilm formation of *C. albicans* strain SC5314 and *C. auris* strain 0390. Each of the approximately 6000 compounds in the library, which includes FDA-approved drugs and compounds at different stages of clinical trials [16], was screened at a final concentration of 20 μM. The primary screenings against both *Candida* species were performed using new 384-well microtiter plates that we adapted from our previously described methodology using 96-well microtiter plates [19]. Two sets of compounds from the library (one for each species) were pre-spotted at the appropriate volumes in individual wells of the 384-well microtiter plates, which were then shipped to our laboratory as assay-ready, bar-coded plates. The newly developed 384-well microtiter plate protocol significantly reduced the amount of time and quantity of compound required for the screening, thereby allowing for a true high-throughput screening of the library. After the screening was completed, the percentage of inhibition for each drug or compound was calculated. Figure 1A,B depict the graphical representation of the results from these primary screenings for *C. albicans* and *C. auris* respectively. Considering the fact that we screened the library at a relatively high concentration of 20 µM, we arbitrarily set up a threshold of 70% inhibition or higher for the identification of hit compounds. Using this criterion, a total of 57 compounds were identified as inhibitors of *C. albicans* SC5314 biofilm formation, and 33 compounds were identified as capable of inhibiting biofilm formation in *C. auris* strain 0390, resulting in initial hit rates of 0.90% for *C. albicans* and 0.55% for *C. auris*, which is in agreement with the fact that *C. auris* biofilms normally display higher levels of resistance compared to their *C. albicans* counterparts [11].

### 3.2. Concentration-Dependent Assays to Confirm “Hits” from the Initial Screen and Establish Their Potency

Next, we performed concentration-dependent assays to confirm and establish the potency of the initial hit compounds. These assays were performed using the same 384-well microtiter plate model, except that each compound was tested at 10 different concentrations (serial dilutions), ranging from 40 µM to 0.078 µM. Two sets of concentration dependent plates, one for *C. albicans* original hits and a second one for *C. auris* hits, were prepared by the Broad Institute after identifying the hits from each initial screen in the Repurposing-Hub-accompanying database and shipped to us for processing. The yeast cell suspensions were added to wells of these plates according to the plate maps, and the plates incubated for 24 h to allow for biofilm formation. The extent of biofilm formation was calculated based on XTT-colorimetric readings, the percentage of inhibition was calculated, and the results were normalized and analyzed to calculate the IC_50_ value for each compound tested. As representative examples, the results of these assays for several initial hits showing dose-responsive inhibitory activity against *C. auris* biofilm formation are provided in Supplementary Figure S1.

In the case of *C. albicans*, a total of 56 of the original 57 “hits” were confirmed (Table 1), for a 98.2% confirmation rate. The accompanying Repurposing Hub database was used to uncover the identity of the confirmed “hits”, which had a variety of original therapeutic indications: 12 were known antifungals, 18 were antiseptics or antibacterials, and 25 (representing 24 unique compounds) had different primary therapeutic indications (i.e., channel blockers, cytokine inhibitors, enzyme inhibitors, etc.) and could represent repositionable candidates as antifungals. Likewise, a total of 30 of the original 33 “hits” from the *C. auris* screen were also confirmed (Table 2), resulting in a 90.9% confirmatory rate. Of these confirmed “hits”, 7 were antifungals or fungicides, 12 were antiseptics or antibacterials, and 11 were potentially repositionable compounds. Overall, there were a total of 19 compounds which were hits in both the *C. albicans* and the *C. auris* screenings (Supplementary Figure S2), of which 5 were classified as antifungals, 8 were antiseptics, and 6 considered to be “repositionable” compounds.

We note that, although the main focus of this particular screen was to identify compounds that can be potentially repurposed as antifungals, the “existing” antifungal hits helped to confirm the reliability and accuracy of this screen, while, at the same time, antiseptic hits can perhaps identify potential compounds that can prevent or eliminate the contamination of skin and/or environmental surfaces with *C. auris* [6,7]. For example, benzethonium chloride, hexachlorophene, and ciclopirox were previously identified by our group in the screen of the Prestwick Chemical Library [25] as effective antiseptics or antibacterials against *C. auris*. The potent antibiofilm and antifungal activity of alexidine has been described before in screens of the Prestwick library [26,27]. In another screen of the Calibr ReFRAME library against *C. auris* 0390 biofilm formation by our lab, cycloheximide was also identified as having efficacy in the inhibition of biofilm formation [28]. Hexachlorophene and thonzonium were identified recently as having broad-spectrum activity against *Aspergillus calidoustus*, *Fusarium oxysporum*, *Fusarium solani*, *Rhizopus oryzae*, *Lomentospora prolificans*, and *Lichtheimia corymbifera*, which are highly resistant fungi [29].

Since the main objective of these particular screens was to identify compounds with novel inhibitory activity against *Candida* biofilms, we focused our attention in those drugs with original therapeutic indications other than antifungals or antiseptics, which are designated in Table 1 and Table 2 as “repositionable compounds”. Several of these drugs, particularly those active against both species, merit some further discussion. Toremifene is a selective estrogen receptor modulator that is used in the treatment of estrogen-receptor-positive breast cancer, and it is thought that this compound exhibits antiestrogen behavior in order to inhibit tumor growth [30,31]. Semapimod is known as an anti-inflammatory drug that suppresses inflammatory cytokine production [32]. It has undergone phase II clinical trials for efficacy in the treatment of Crohn’s disease; however, it has yet to move forward into phase III [33]. Plurisin #1 is a pluripotent cell-specific inhibitor that induces apoptosis, and it prevents undifferentiated cells from developing into tumors when tissues are regenerated [34]. Darapladib is known to inhibit lipoprotein-associated phospholipase A2, an indicator of atherosclerosis in patients with coronary heart disease [35]. Although this compound went into phase III clinical trials, it was not able to reduce the risk of cardiovascular events in patients with chronic coronary heart disease; thereby, its development was discontinued for this purpose [36]. Tribomsalan is a photosensitizing agent that was previously used in over-the-counter drugs and cosmetic products, but it has since been removed because photosensitizing agents are thought to cause higher risk of non-melanoma skin cancer [37]. KHK-IN-1 is a ketohexokinase inhibitor, but it has so far not been approved to treat any specific disease [38]. Bithionol is an antihelminthic, especially used in treating liver flukes, and it has been reported to have antibacterial activity [39]. This compound was previously used in topical products; however, it was removed from the market in the U.S. because of skin disorders that occurred, although it is currently used in other countries to treat different types of helminth infections [40]. Although its removal from the U.S. market presents a problem for future repositioning, this drug could be used perhaps to coat medical equipment like catheters to prevent the growth of *C. auris*.

As seen in Table 1 and Table 2, we identified rapamycin (also referred to as sirolimus) and different rapalogs as the drugs displaying the lowest IC_50_ value (approaching the picomolar range) among all confirmed compounds. This is not surprising, as rapamycin is a highly potent immunosuppressant also known to display both antifungal and antineoplastic properties [41,42,43]. Temsirolimus, a common hit to both *C. albicans* and *C. auris* screens, is currently approved to treat renal cell carcinoma, a type of kidney cancer [44,45], whereas zotarolimus, which was a hit against *C. auris* only, is used in drug-eluting stents to treat cardiac restenosis, the narrowing of blood vessels [46]. We recently reported on another rapalog, everolimus, as our main leading repositionable compound from the Pandemic Response Box, also with potent biofilm-inhibitory activity against these two *Candida* species [47]. Temsirolimus is actually an ester analog of rapamycin, and, after administration in humans, it is converted to its major metabolite (rapamycin) via enzymatic hydrolysis, which results in improved solubility and pharmacokinetic properties [48,49]. Like rapamycin, temsirolimus is also a highly specific inhibitor of the mammalian target of rapamycin (mTOR), which has been implicated in multiple tumor-promoting intracellular signaling pathways [50,51]. Temsirolimus was the first mTOR inhibitor to be approved as an anticancer agent; more specifically, it was approved by the FDA for the treatment of advanced renal cell carcinoma in May 2007 [52], and displays promising activity in other cancers, including lymphomas, as well as breast, endometrial, and neuroendocrine cancers [53]. Interestingly, temsirolimus seems to display much more potent antifungal activity compared to other rapamycin analogs (“rapalogs”) such as tacrolimus (FK 506) [47]. Much less is known about the NFκB inhibitor BAY 11-7082 (identified as a hit in both *C. albicans* and *C. auris* screens) and its analog BAY 11-7085 (a hit in the *C. albicans* screen only), which were also among the top leading repositionable candidates. In mammalian cells, they inhibit IκB-α phosphorylation and are known to regulate cytokine function and, specifically, inflammation [54]; as a result, they exhibit broad-spectrum anti-inflammatory activity against multiple targets [55]. They display pharmacological activities that include anticancer, neuroprotective, and anti-inflammatory effects, but have been primarily used as a bioactive small molecule for gene regulation research [54,55]. BAY 11-7082 has also undergone preclinical studies to examine its effect in preventing inflammation after hematopoietic stem cell transplants in mice, its anti-tumor activity, and its efficacy in protecting against psoriasis [56,57,58]. Interestingly, compounds BAY 11-7082 and BAY 11-7085 were among our top hits in a screening of Sigma’s LOPAC library for inhibitors of *C. albicans* biofilm formation [59], and were also among the main hits reported by Watamoto et al. when they screened the same LOPAC library for antifungal activity against *C. albicans* [60]. Escobar et al. reported on the activity of BAY 11-7085 against *C. albicans* single- and mixed-species biofilms (i.e., Staphylococcus aureus and *C. albicans*) [61], and, most recently, both BAY 11-7085 and BAY 11-7082 were identified as inhibitors of *C. albicans* filamentation during a high-content imaging screen [62]. From our initial screening and concentration-dependent assays, among these two compounds, BAY 11-7082 displayed higher efficacy (maximum percent inhibition) and potency (lower IC_50_), and was selected for follow-up studies.

In addition, the antifungal activity of ebselen, which was one of the main hits against *C. auris* only, has been previously reported by our lab [25] and others [63]. We identified this organoselenium compound as a highly effective inhibitor of planktonic growth of *C. auris* 0390 in a screen of the Prestwick Chemical Library, with follow-up studies indicating its activity against all other *C. auris* strains in the CDC panel, several other *Candida* spp., and different pathogenic fungi [25]. Ebselen is known to have anti-inflammatory and anti-oxidant properties, and it acts as a glutathione peroxidase mimic, which allows it to prevent damage from reactive oxygen species [64]. It has been through phase II clinical trials for its ability to prevent noise-induced hearing loss [65] as well as other diseases. In spite of these trials, ebselen has not yet been approved for the treatment of any disease.

Thereby, we selected temsirolimus, ebselen, and compound BAY 11-7082 (Supplementary Figure S3) as our main repositionable compounds for follow-up studies characterizing their in vitro and in vivo antifungal activity.

### 3.3. Determination of the Spectrum of Antifungal Activity of the Leading Repositionable Compounds Ebselen, Temsirolimus, and BAY 11-7082

Once their anti-biofilm activity against *Candida* spp. was fully established, we were interested in testing the activity of the selected leading repositionable compounds temsirolimus and BAY 11-7082 against a panel of medically important fungi, and, in doing so, determining their spectrum of antifungal activity. We have previously reported on similar experiments with ebselen, at a much more limited scale [25], and we wanted to expand our investigations into the spectrum of its antifungal activity here. These assays were performed by the Fungus Testing Laboratory utilizing standardized CLSI methodologies for antifungal susceptibility testing against yeasts and molds [20,21], with MICs determined at both 50% and 100% inhibitory endpoints. Table 3 and Table 4 summarize the in vitro activity of the selected compounds against yeasts and filamentous fungi.

As previously indicated by our group [25], these results confirmed the relatively broad spectrum of antifungal activity of ebselen against yeasts and filamentous fungi, perhaps with the exception of the Mucorales. As seen in the tables, the MIC values determined for temsirolimus against different species of yeasts indicate the potent antifungal activity of this rapalog against all clinical isolates belonging to the different species of *Candida*, including *C. albicans*, *C. auris, C. parapsilosis*, *C. glabrata*, and *C. krusei*, irrespective of their fluconazole resistance. Importantly, these inhibitory concentrations are within the range of the clinically achievable concentration of the drug in blood from patients treated with a conventional dosing regimen of temsirolimus [49]. However, temsirolimus showed limited antifungal activity against Cryptococcus neoformans, with MIC values of 1 μg/mL detected for most isolates when using the 50% reading endpoint, but MIC > 32 μg/mL (the highest concentration used in these assays) for all clinical isolates tested when using the 100% reading endpoint. Likewise, all filamentous fungi tested were not inhibited by temsirolimus, with MIC > 32 μg/mL for all clinical isolates tested belonging to different species of molds when using the 100% reading endpoint, although some lower MIC values when using the 50% reading endpoint seem to indicate some the limited antifungal activity of this rapalog against molds. In contrast, BAY 11-7082 displayed a remarkable broad spectrum of antifungal activity with potent in vitro activity against both yeasts and filamentous fungi. MIC values ranging from 0.25 to 4 μg/mL (at 100% inhibition) were detected for all yeast clinical isolates tested, including all different *Candida* spp. and *C. neoformans*, which compared favorably to fluconazole MICs. But perhaps most interesting is the fact that BAY 11-7082 seems to display potent activity also against all species of filamentous fungi tested, including the Mucorales (both Rhizopus and Mucor species), *Aspergillus* spp., *Scedosporium* spp., *Lomentospora prolificans*, *Fusarium* spp., *Alternaria*, *Curvularia,* and *Exserohilum* spp., with MICs which, in most instances, compared quite favorably with their corresponding MIC values for voriconazole and/or posaconazole. Of note, infections caused by non-Aspergillus molds are becoming increasingly frequent and are difficult to treat, as many of these species are remarkably recalcitrant to most current existing antifungals, often leading to very poor outcomes in patients suffering from these devastating infections [24]. As such, developing antifungals with activity against these filamentous fungi represents one of the most pressing needs in the field of medical mycology.

### 3.4. In Vivo Efficacy of Ebselen, Temsirolimus, and BAY 11-7082 in the Murine Models of Hematogenously Disseminated Candidiasis

**Figure 2:**
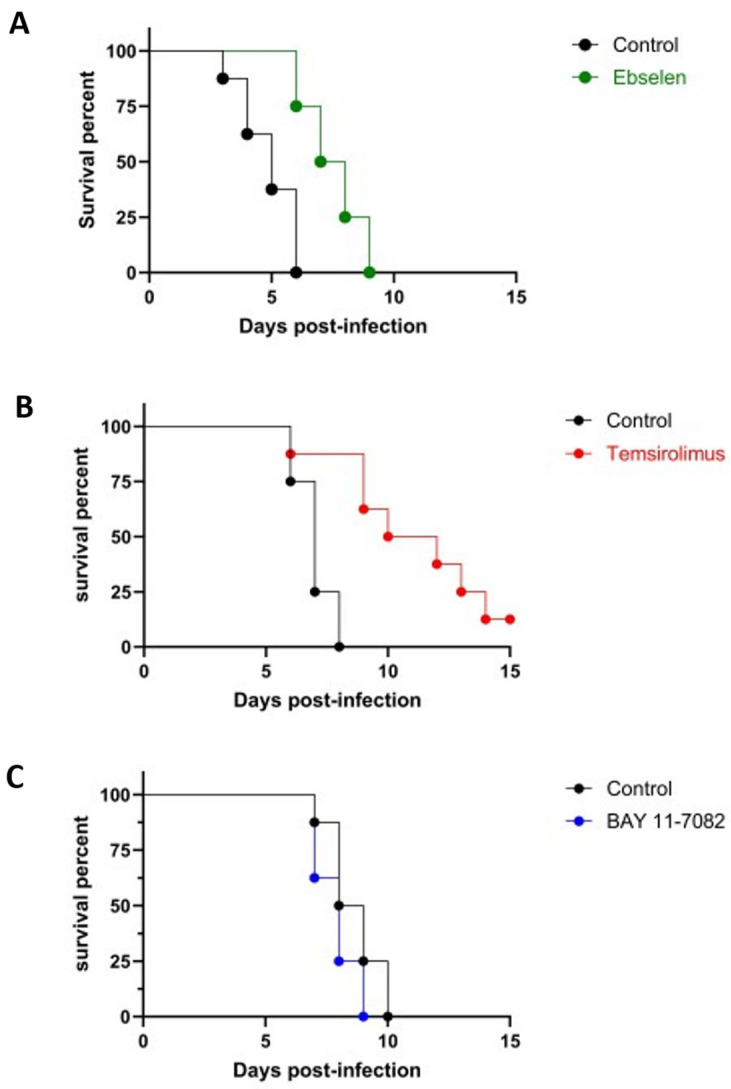
Evaluation of protective effects of treatment with ebselen (**A**), temsirolimus (**B**), and compound BAY 11-7082 (**C**) in the murine model of hematogenously disseminated infection by *C. albicans*.

In an initial set of experiments, we proceeded to preliminarily examine the potential in vivo antifungal activity of ebselen, temsirolimus, and compound BAY 11-7082, for which we used the well-established mice model of hematogenously disseminated *C. albicans*. The resulting survival curves are shown in Figure 2. Treatment with ebselen and temsirolimus increased the median survival of animals infected with *C. albicans* compared the untreated control group, and these differences were statistically significant (*p* = 0.0011 and *p* = 0.0072, respectively). In contrast, under the specific parameters (i.e., infecting inoculum, dose, regimen, etc.) used in this set of experiments, mice treated with compound BAY 11-7082 did not exhibit any significant differences in survival compared to the control group. Although these represent preliminary results, this lack of activity in vivo, as opposed to its potent antifungal effects in vitro, points potentially to the need to improve the drug-like properties of this class of compounds [55].

**Figure 3:**
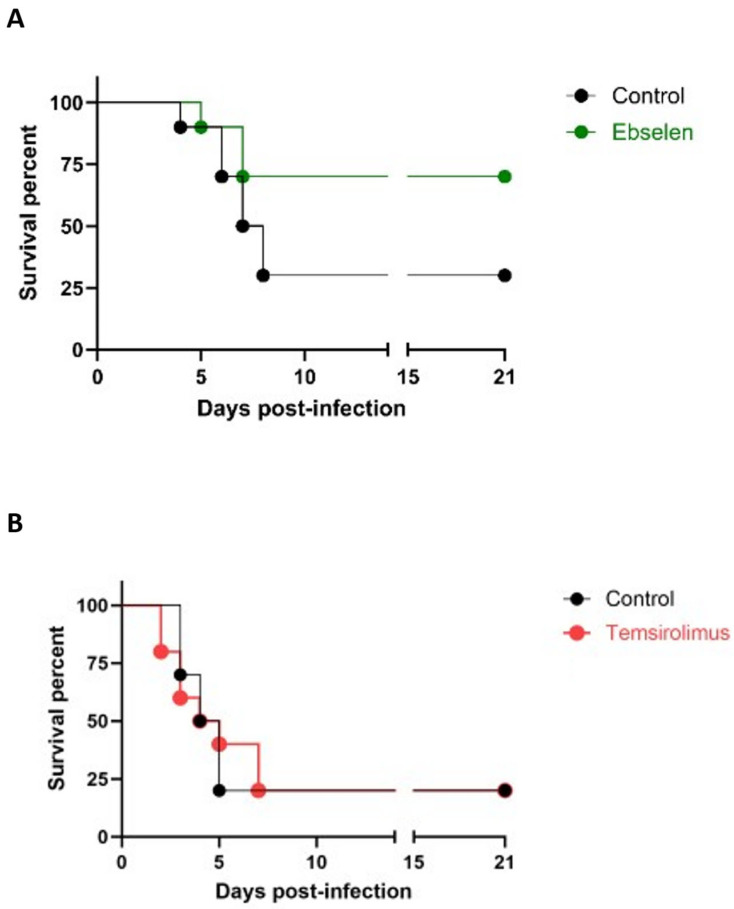
Evaluation of protective effects of treatment with ebselen (**A**) and temsirolimus **(B**) in the murine model of hematogenously disseminated infection by *C. auris*.

Having demonstrated their in vivo activity in the *C. albicans* model, we then proceeded to evaluate the protective effects of ebselen and temsirolimus treatment in the murine model of hematogenously disseminated candidiasis caused by *C. auris*. As shown in Figure 3A, treatment with ebselen increased the median survival of animals from 7.5 days (for untreated control) to over 21 days. When the resulting survival curves were analyzed, there was a trend towards improved survival against *C. auris* infection, although the differences did not achieve statistical significance (*p* = 0.0645). Moreover, under the specific conditions used in this set of experiments, we were unable to detect any protective effects of treatment with temsirolimus, with the resulting survival curves virtually overlapping those obtained for the control (untreated) group (Figure 3B). Compared to the *C. albicans* model, this is a more demanding model since it uses immunosuppressed mice [22,23], which could be partially responsible for the more limited protective effects observed in this model. In future experiments, further assessment of the potential protective effects of treeatment with ebselen and temsirolimus in this model may involve the evaluation of different parameters of both the infection (i.e., infecting inocula) and treatment (i.e., dose, frequency and route of administration, etc.).

In summary, this screen identified compounds in the Repurposing Hub library that inhibited 70% or more of biofilm formation by *C. albicans* and/or *C. auris*. Besides known antifungals and antiseptics, several other drugs were identified, with a variety of original therapeutic indications, modes of action, and clinical trial records. Further in vitro and in vivo characterization of the antifungal activity was performed for the leading repositionable compounds ebselen, temsirolimus, and BAY 11-7082. It is likely that some of these compounds have direct effects on the fungal cell, presumably “off-target” relative to their original clinical indications or biological activities on human cells. Despite some promising initial results, further experiments are needed to evaluate and confirm their promise to be repurposed as antifungal drugs for the prevention and treatment of *C. albicans* and *C. auris* infections, as well as for potentially other fungal infections, for which there is a dire and urgent need.

## Figures and Tables

**Table 1:** Identity and biofilm-inhibitory activity of confirmed hit compounds against *C. albicans* SC314. Information for each compound includes percent inhibition during primary screen, as well as maximum level of inhibition (efficacy) and calculated IC_50_ values (potency) from concentration-dependent confirmatory experiments.

Compound Name	% Inhibition Initial Screen	Maximum % Inhibition from Dose–Response Assays	IC_50_ (µM) from Dose–Response Assays
Antifungals/Fungicides			
Anidulafungin	92.32	99.18	<0.078
Cerulenin	98.27	96.08	4.625
Ciclopirox	93.51	91.77	1.200
Flucytosine	86.81	80.51	2.936
JIB04	74.9	76.2	21.220
Oligomycin-a	73.23	72.00	5.131
Sertaconazole	81.89	68.02	4.687
Sulconazole	71.52	73.51	0.114
Terbinafine	71.14	68.89	22.400
Terconazole	69.58	69.37	1.304
Toyocamycin	98.45	95.38	1.582
Voriconazole	70.42	65.32	<0.078
Antiseptics/Antibacterials			
Alexidine	98.69	99.59	1.246
Benzethonium	99.62	100	2.436
Benzyldimethylhexadecylammonium	98.58	99.88	18.180
Bithionol	84.19	81.24	7.344
Brilliant-green	99.29	96.25	1.183
Cetrimonium	99.01	99.59	4.759
Cetylpyridinium	100.42	99.77	6.073
Chlorhexidine	98.91	98.42	11.520
Chloroxine	91.52	97.49	2.273
Clioquinol	89.7	84.5	0.871
Crystal-violet	98.65	96.47	1.346
Dequalinium	98.15	96.47	15.440
Domiphen	99.94	97.14	12.880
Octenidine	97.93	95.02	9.014
Oritavancin	81.94	83.87	15.460
Phenylmercuric acetate	99.89	98.38	<0.078
Thiomersal	99.53	96.86	0.448
Triclosan	98.65	98.48	6.220
Repositionable Compounds			
Anisomycin	85.08	82.3	2.973
Atiprimod	72.38	73.22	19.450
BAY 11-7082	99.67	98.01	6.733
BAY 11-7085	98.84	96.81	12.180
Broxaldine	93.36	88.35	1.708
Ceritinib	96.09	83.04	18.650
Clomifene	79.4	100	9.279
Darapladib	99.16	99.12	18.640
Enasidenib	89.91	99.5	11.710
Fingolimod	72.69	93.11	9.600
K145	96.85	96.43	18.420
NSC-319726	90.59	92.05	1.993
Otilonium	97.28	97.43	6.750
Pinaverium	76.42	75.02	25.970
Pitavastatin	79.18	95.24	4.015
Plurisin-#1	74.23	70.88	27.760
Sanguinarium-chloride	97.85	96.96	4.928
SC-144	77.72	84.39	9.163
Semapimod	98.83	98.71	9.055
Sirolimus *	80.96	88.30	0.606
Sirolimus *	73.73	85.61	0.614
Temsirolimus	71.54	84.43	0.376
Toremifene	81.49	84.88	4.952
Triclabendazole	70.53	62.4	15.740
U-18666A	97.78	95.35	4.792

* Indicates Sirolimus from two different sources represented in the library and tested independently.

**Table 2:** Identity and biofilm-inhibitory activity of confirmed hit compounds against *C. auris* strain 0390. Information for each compound includes percent inhibition during primary screen, as well as maximum level of inhibition (efficacy) and calculated IC_50_ values (potency) from concentration-dependent confirmatory experiments.

Compound Name	% Inhibition Initial Screen	Maximum % Inhibition from Dose–Response Assays	IC_50_ (µM) from Dose–Response Assays
Antifungals/Fungicides			
Anidulafungin	77.09%	95.08%	3.621
Cerulenin	82.94%	87.71%	30.640
Ciclopirox	77.78%	77.67%	9.779
Flucytosine	77.25%	74.40%	10.600
Ketoconazole	76.00%	57.90%	10.720
Tavaborole	91.78%	96.11%	1.075
Terconazole	75.64%	60.70%	7.146
Antiseptics/Antibacterials			
Alexidine	97.69%	87.30%	3.610
Benzethonium chloride	104.00%	89.55%	8.898
Brilliant-green	88.94%	88.08%	4.603
Cetylpyridinium	100.19%	81.16%	17.060
Crystal-violet	82.26%	93.86%	2.707
Cycloheximide	73.54%	86.69%	2.574
Hexachlorophene	81.50%	95.90%	4.989
Octenidine	94.32%	77.06%	8.423
Phenylmercuric acetate	102.10%	95.49%	0.210
Thiomersal	92.70%	92.83%	0.541
Thonzonium	101.31%	91.40%	8.406
Triclosan	98.40%	77.83%	5.020
Repositionable Compounds			
BAY 11-7082	96.07%	93.24%	17.460
Bithionol	89.07%	74.40%	5.367
Darapladib	90.05%	82.38%	18.890
Ebselen	95.21%	82.59%	16.910
KHK-IN-1	98.80%	94.47%	37.140
Plurisin-#1	81.30%	73.37%	38.860
Semapimod	90.63%	96.31%	11.370
Temsirolimus	98.62%	91.40%	0.965
Toremifene	90.00%	96.09%	14.470
Tribomsalan	69.57%	62.93%	25.560
Zotarolimus	75.83%	90.58%	2.777

**Table 3:** MIC values of the leading repositionable compounds ebselen, temsirolimus, and BAY 11-7082 against multiple clinical isolates belonging to different species of yeast, in comparison to fluconazole. Values are in μg/mL.

Species	Isolate	Ebselen		Temsirolimus		BAY 11-7082		Fluconazole
		50%	100%	50%	100%	50%	100%	50%
*C. parapsilosis* QC	ATCC 22019	0.5	2	1	1	4	8	1
*C. krusei* QC	ATCC 6258	1	4	1	2	0.5	0.5	16
*C. albicans*	ATCC 90028	1	2	1	1	1	2	0.25
	SC5314	2	2	1	1	1	2	≤0.125
	Ca-1	1	2	1	1	1	1	0.5
*C. auris*	Cau-1	0.125	0.25	1	1	2	4	>64
	Cau-2	0.125	1	1	1	0.25	1	>64
	Cau-3	0.25	0.25	1	1	2	4	2
*C. glabrata*	Cg-1	1	2	0.5	1	1	2	64
	Cg-2	1	2	0.5	1	1	2	4
	Cg-3	0.5	2	1	1	0.5	1	0.5
*C. parapsilosis*	Cp-1	0.25	2	1	1	2	4	0.5
	Cp-2	0.25	2	0.5	1	2	4	0.25
	Cp-3	0.5	2	1	1	2	4	0.5
*Cryptococcus neoformans*	Cn-1	2	2	1	>32	1	1	64
	USC1597	2	4	1	>32	1	1	4
	H99	2	2	1	>32	1	1	16

**Table 4:** MIC values of the leading repositionable compounds ebselen, temsirolimus, and BAY 11-7082 against multiple clinical isolates belonging to different species of filamentous fungi, in comparison to voriconazole and/or posaconazole. Values are in μg/mL.

Species	Isolate	Ebselen		Temsirolimus		BAY 11-7082		Voriconazole	Posaconazole
		50%	100%	50%	100%	50%	100%	100%	100%
*P. variotii* QC	MYA-3630	4	4	16	>32	1	1	0.125	≤0.03
*Rhizopus arrhizus*	Rh-1	4	>32	>32	>32	4	4	-	1
	Rh-2	8	>32	>32	>32	4	4	-	0.5
	Rh-3	2	>32	>32	>32	0.5	1	-	0.5
*Mucor* spp.	Mu-1	8	>32	>32	>32	4	4	-	2
	Mu-2	8	>32	>32	>32	2	4	-	1
	Mu-3	4	>32	0.125	>32	2	4	-	2
*Aspergillus flavus*	ATCC204304	4	4	>32	>32	4	4	1	-
	Afl-1	4	4	>32	>32	2	4	1	-
	Afl-2	4	4	>32	>32	2	4	1	-
*Aspergillus fumigatus*	AF293	4	4	>32	>32	1	1	0.5	-
	Af-1	4	4	>32	>32	1	1	>16	-
	Af-2	4	4	>32	>32	1	1	4	-
*Fusarium* spp.	Fu-1	4	4	2	>32	1	1	>16	-
	Fu-2	4	8	2	>32	0.5	1	>16	-
	Fu-3	4	8	1	>32	1	2	>16	-
*Lomentospora prolificans*	Sc-1	4	8	0.5	>32	0.5	0.5	>16	-
*Scedosporium* spp.	Sc-2	4	4	1	>32	0.5	0.5	2	-
	Sc-3	2	2	0.5	>32	0.25	0.5	1	-
*Altenaria*	Al-1	0.5	2	>32	>32	0.5	1	1	-
*Curvularia*	Cu-1	1	2	16	>32	0.5	1	0.5	-
*Exserohilum*	Ex-1	1	4	16	>32	0.5	1	2	-

## Data Availability

Most of the data are contained within the article or Supplementary Material. Data from primary screenings and dose–response experiments are available from the corresponding author upon request.

## Supplementary Materials

The following supporting information can be downloaded at: https://www.mdpi.com/article/10.3390/jof9090879/s1. Figure S1: Representative results of concentration-dependent experiments to confirm the activity and establish the potency of initial hits against *C. auris* biofilm formation. Figure S2: Venn diagram depicting numbers of confirmed inhibitory compounds against either or both *C. albicans* and *C. auris* biofilm formation. Figure S3: Chemical structures and properties of the leading repositionable compounds ebselen (A), temsirolimus (B), and compound BAY 11-082 (C).
