# Supplementary material for: High-Throughput Screening of the Repurposing Hub Library to Identify Drugs with Novel Inhibitory Activity against Candida albicans and Candida auris Biofilms
Source: J Fungi (Basel). 2023 Aug 27;9(9):879. doi: 10.3390/jof9090879 (PMC10532723; doi:10.3390/jof9090879)
Supplement: Supplementary file 1 [file jof-09-00879-s001.zip › jof-2552566-supplementary.pdf]

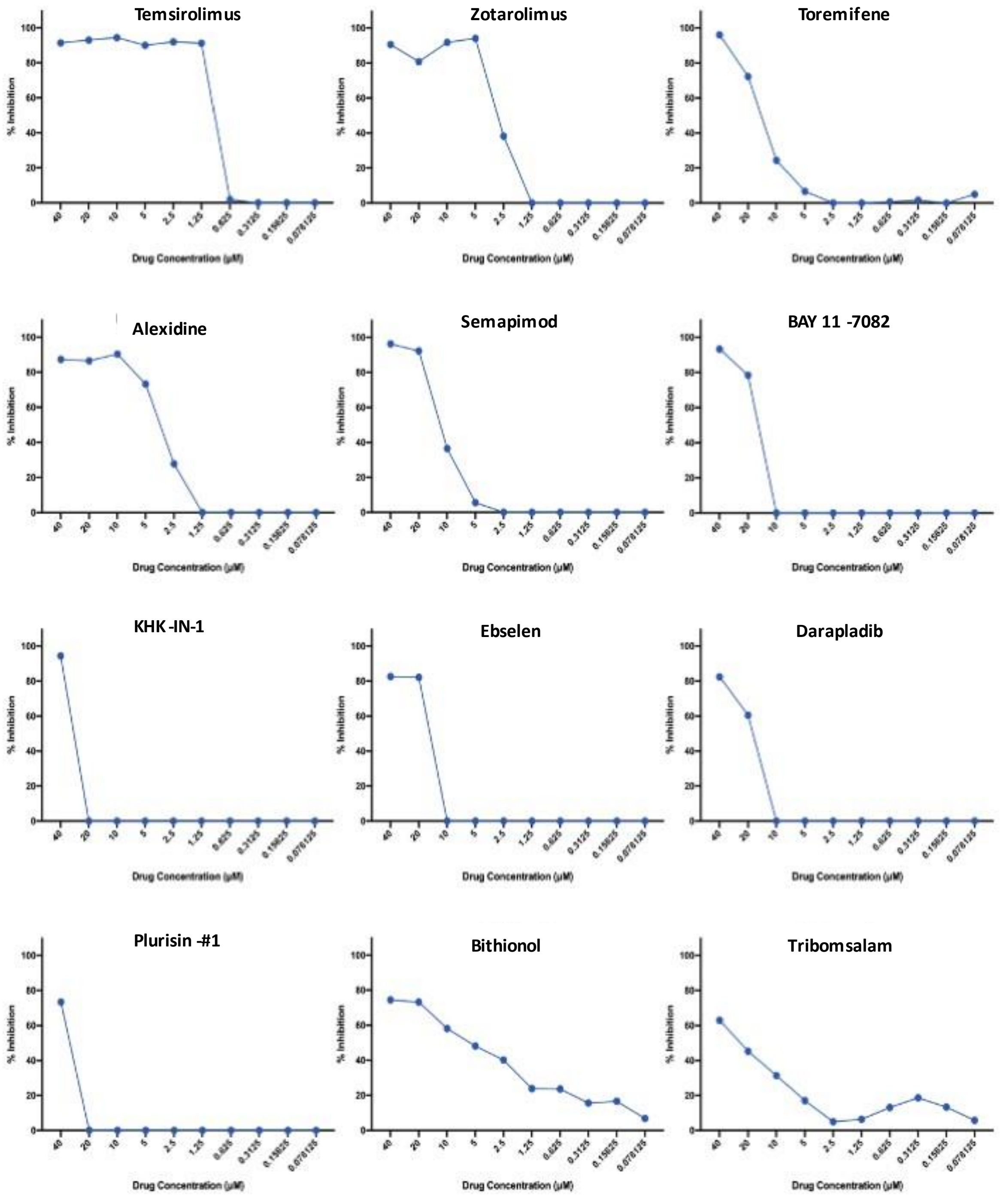

Figure S1: Representative examples of results from concentration-dependent confirmatory measurement. Graphs show the 10-point concentration curves for repositionable hits from the primary screening showing dose-responsive *C. auris* biofilm inhibition, by plotting the percent biofilm inhibition achieved at each concentration tested. From these results, their IC_{50} was calculated as reported in Materials and Methods.

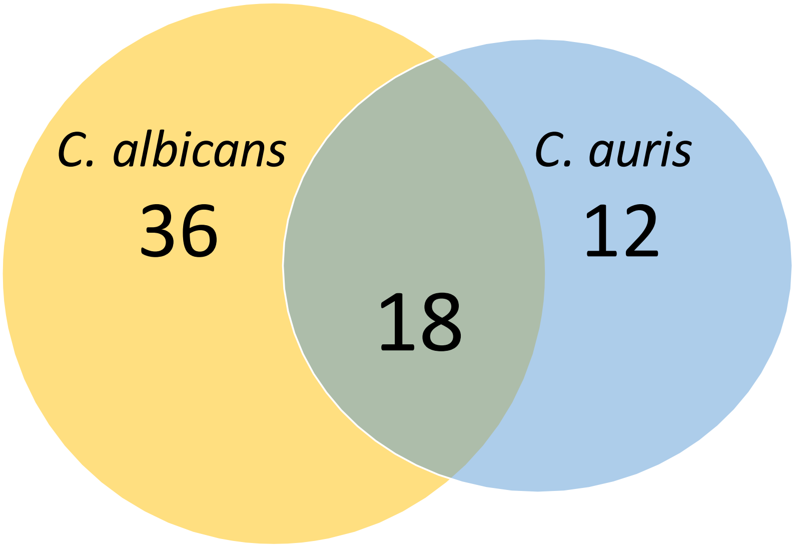

Figure S2: Venn diagram depicting numbers of confirmed inhibitory compounds against either or both *C. albicans* and *C. auris* biofilm formation.

A

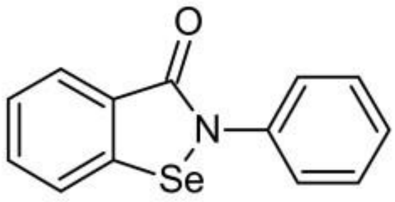

B

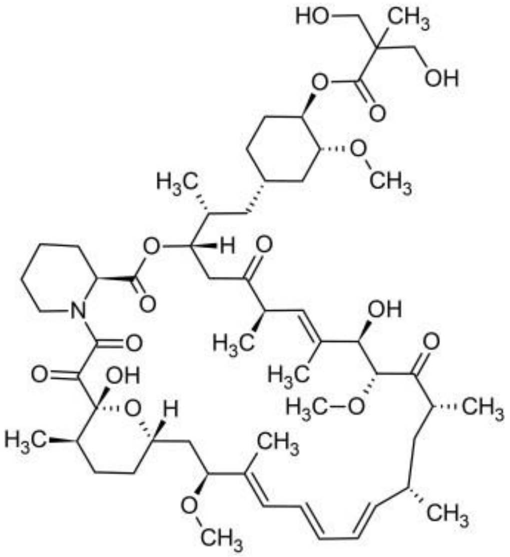

C

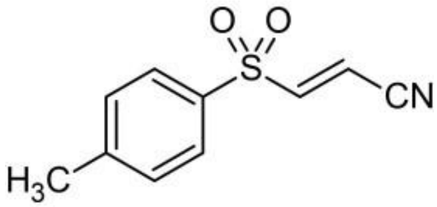

Figure S3: Chemical structures and properties of the leading repositionable compounds ebselen (**A**), temsirolimus (**B**) and compound BAY 11-082 (**C**)
